# Modular Continuum Manipulator: Analysis and Characterization of Its Basic Module

**DOI:** 10.3390/biomimetics3010003

**Published:** 2018-02-14

**Authors:** Anand Kumar Mishra, Alessio Mondini, Emanuela Del Dottore, Ali Sadeghi, Francesca Tramacere, Barbara Mazzolai

**Affiliations:** 1 Center for Micro-BioRobotics, Istituto Italiano di Tecnologia, 56025 Pontedera, Italy; emanuela.deldottore@iit.it (E.D.D.); ali.sadeghi@iit.it (A.S.); 2 The BioRobotics Institute, Scuola Superiore Sant’Anna, 56025 Pontedera, Italy

**Keywords:** continuum manipulator, soft robot, modular arm, compliant structure, large deformation, constant curvature, tendon-driven actuation, kinematic modeling, planar spring, beam theory

## Abstract

We present the basic module of a modular continuum arm (soft compliant manipulator for broad applications (SIMBA)). SIMBA is a robotic arm with a hybrid structure, namely a combination of rigid and soft components, which makes the arm highly versatile, dexterous, and robust. These key features are due to the design of its basic module, which is characterized by a three-dimensional workspace with a constant radius around its rotation axis, large and highly repeatable bending, complete rotation, and passive stiffness. We present an extensive analysis and characterization of the basic module of the SIMBA arm in terms of design, fabrication, kinematic model, stiffness, and bending behavior. All the theoretical models presented were validated with empirical results. Our findings show a positional typical error of less than ≈6% in module diameter (highly repeatable) with a passive stiffness of 0.8 N/mm (≈1 kg load). Our aim is to demonstrate that this kind of robotic element can be exploited as an elementary module of a more complex structure, which can be used in any application requiring high directional stiffness but without the need for an active stiffness mechanism, as is the case in daily activities (e.g., door opening, water pouring, obstacle avoidance, and manipulation tasks).

## 1. Introduction

**Figure 1:**
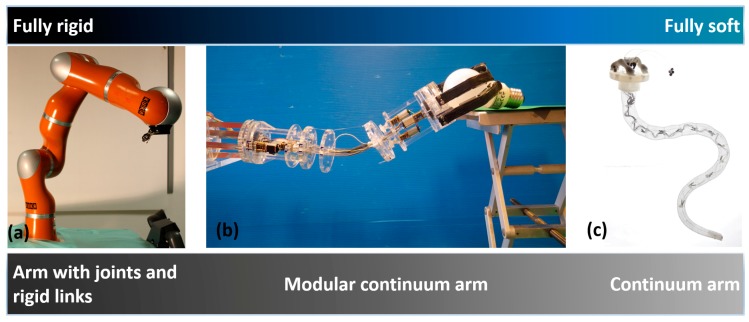
Three examples of arms with different features in terms of materials and stiffness. (**a**) The KUKA arm is a fully rigid structure with joints and rigid links [15]; (**b**) the soft compliant manipulator for broad applications (SIMBA) arm is a modular continuum arm with a hybrid structure with rigid and soft materials [16]; (**c**) the soft robot arm inspired by the octopus is a completely soft continuum arm [8,17]. (**c**) Image published under the Creative Commons Attribution (CC BY) license and reproduced from [17].

In the early 1950s, the first robotic solutions were developed in order to address repetitive tasks requiring precision, strength, and endurance (e.g., car automation, food packaging, industrial welding, and so on) [1]. These autonomous solutions consisted of rigid links driven by inverse kinematics and were conceived to work in a structured space. The rigid structure of the arm guaranteed the robustness and stiffness required for high payload activities; whereas the structured space enabled them to be easily controlled and highly precise. However, these robots have various limitations: difficulty in handling fragile objects, potentially highly dangerous unintentional human–robot interactions, and the inability to work in unstructured environments [2]. These issues represent some of the biggest challenges that researchers have faced in recent years. Specifically, the main aim focus on the conception and development of technologies capable of compliant intentional and unintentional interactions with items (including humans). Soft robotic solutions are one of the most promising approaches as they are highly compliant and have dexterous arms (see Figure 1). These solutions should guarantee safe item–robot interaction [3,4,5], as well as enabling the robot to move in unstructured environments, confined spaces, etc. These manipulators, which are called soft continuum arms, are mostly designed with a continuum architecture without any joints and links [6]. 

To develop such a soft continuum arm, the commonly used actuators are flexible fluidic actuation, McKibben actuators, tendon-motor actuators or shape memory alloys (SMAs) [6,7]. The octopus, which is characterized by a completely soft structure, has inspired several works using different actuations [8,9,10,11]. Other examples, such as Festo’s Bionic Handling Assistant arm [12], uses bellows as actuators. STIFF-FLOP [13], a variable stiffness module for surgical application, is actuated pneumatically; and for spatial applications, flexible fluidic elastomers have been exploited [14].

Although soft continuum arms show high dexterity, adaptability, and conformability to the surrounding external environment they are often not able to achieve a high stiffness and robustness. A solution capable of providing the robustness of rigid arms and the versatility of soft arms still remains to be found.

In order to address such an issue, attempts have been made by exploiting hybrid structures, characterized by a combination of soft and rigid elements. Some interesting results have been obtained such as: the Air-Octor arm which uses a combination of a pneumatic chamber and a tendon-based mechanism for the actuation [18]; a compliant hyper-redundant arm inspired by an elephant trunk [19], where several segments composed of rigid disks are connected and actuated with springs and tendon-based mechanism; a tensor arm [20], where several joints separated by metal disks are actuated by cables and motors; a helical spring-based backbone tendril arm [21]; a robot arm with a compliant structure obtained with notches [22]; and an arm with a flexible backbone separated by rigid disks and actuated with tendons [23]. All these hybrid continuum arms provide better dexterity, conformability, and adaptability compared to completely rigid arms, and provide a better load-bearing capacity, speed, and accuracy compared to completely soft continuum arms [2,24]. However, they still have limited bending capabilities, induced by their rigid internal links or by employing bulky components which also yield high inertia. They are mainly multi-sectioned continuum arms, but none of them show modularity, independent actuation, or relative motion among their sections, limiting in this way the working space and controllability. 

In our work, we developed a new modular hybrid continuum manipulator (soft compliant manipulator for broad applications (SIMBA)), combining rigid and soft components (Figure 1b). SIMBA consists of a reconfigurable gripper, a base slider, and two modules of a fixed diameter (60 mm). The gripper and arm modules are made of soft and rigid components (poly(methyl) methacrylate (PMMA), flat springs, reinforced rubber, and elastic waistband) and a tendon-motor actuation. SIMBA’s design is discussed in [16], focusing on the gripper characterization and an analysis of the manipulation capabilities.

The present paper analyses SIMBA’s basic module in terms of modeling, characterization, and manufacturing. We show how the integration of the actuation in the module facilitates the control (i.e., relative positioning is not affected by the other modules), and at the same time does not affect the compliancy and the bending capabilities of the module itself (the actuation part is limited to a small portion of the module). The passive stiffness and large bending capabilities of the module due to the geometrical benefits of the planar springs are also demonstrated. An arm consisting of these modules can guarantee dexterity and facilitate distributed control.

In the following, Section 2.1 and Section 2.2 outline the design and fabrication of the basic module of the arm, respectively. Section 2.3 and Section 2.4 analyze the kinematics and stiffness from a modeling point of view. Section 2.5 presents the experimental setups to validate the mathematical models. Section 3 and Section 4 discuss results and the performance of the module in terms of stiffness, curvature, working space, and design.

## 2. Materials and Methods

### 2.1. Conceptual Design of SIMBA’s Basic Module

In designing the robot, we aimed to have the following key features: lightweight, modularity, independent actuation, and compliance. We thus combined soft and rigid components to provide enough robustness for weight lifting tasks and adaptability for challenging tasks. We employed planar beams, a tendon-motor-driven actuation, and a combination of bending and rotational joints to enhance movement.

**Figure 2:**
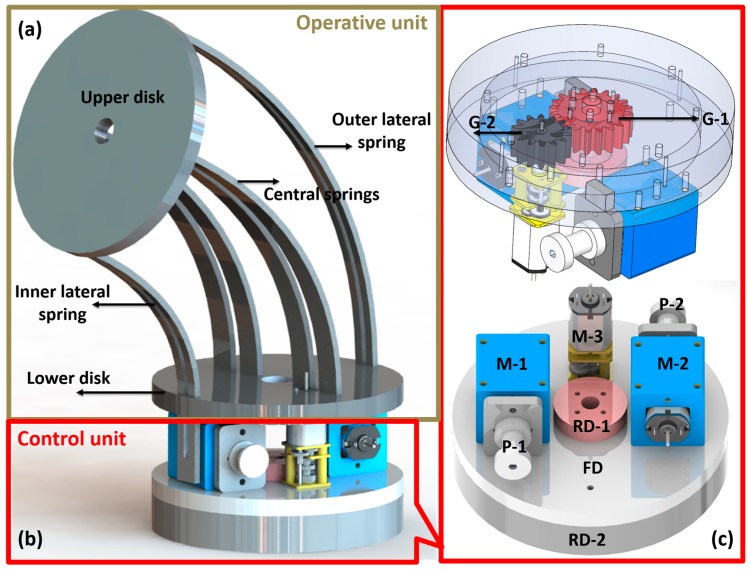
Design of SIMBA’s basic module. (**a**) Operative unit; (**b**) control unit; (**c**) details of the control unit. RD-1= upper rotational disk 1; RD-2 = bottom rotational disk 1; P-1 = pulley for motor 1; P-2 = pulley for motor 2; FD = fixed disk for motor fixing; M-1 = motor bending; M-2 = motor stiffening; M-3 = motor for rotation; G-2 = driving gear connected to M-3; G-1 = pinion gear fixed on RD-2.

The basic module is 60 mm in diameter, 144 mm in length, and 173 g in total weight. It consists of two subsections: an operative unit and a control unit (Figure 2). The first unit is used to perform movements in three-dimensional (3D) space by bending and rotation, while the second unit is used to provide actuation and control of the operative unit. 

The operative unit consists of two disks and six planar springs with a rectangular shape which provide high passive stiffness in one direction. Four planar springs (referred to as the ‘central springs’) are collinearly embedded as the backbone of the module. Two other springs, called ‘lateral springs’, have lateral slots and are connected at the borders of the disks (upper and lower disks of bending unit) parallel to the central springs (see Figure 2a). These two lateral springs are fixed to the upper disk but are free to move linearly on the lower side. They help to reduce buckling and are used to guide the module to maintain constant curvature while bending. 

The actuation unit consists of two rotatory disks (RD-1, RD-2) and one fixed disk (FD) (see Figure 2b,c) dedicated to host three direct current (DC) gear motors, two for bending (M-1, M-2) and one for module rotation (M-3). For the rotational motion, we used two spur gears (G-1, G-2): G-1 is directly connected to the motor and to the FD, and G-2 is a reduction gear train mechanism, as described in the following, fixed on RD-2. 

The proposed module has two degrees of freedom: (1) rotation about its own axis (the module can compute a complete rotation of 360°), and (2) bending on a plane. The module can move in 3D space thanks to the combination of these two movements. Bending is achieved by means of two motors connected to two tendons, allowing side-to-side movements and tuning of the module stiffness in the bending plane. 

### 2.2. Fabrication and Control 

To fabricate the arm module, we used six stainless steel planar springs of 100 × 8 × 0.2 mm (material type-EN 1.4301, flat spring, Misumi Europa GmbH, https://uk.misumi-ec.com/); Young’s modulus = 193 GPa; shear modules = 68 GPa; density 7850 kg/m^3^). The two lateral springs were cut longitudinally which enabled the cables to move freely inside without mechanical interference. To fabricate the supporting structure of the module, we used three different thicknesses (3, 5, and 8 mm) of Derlin (Betametalli, Prato, Italy). Derlin has a tensile strength of 80 MPa, Young’s modulus (E) of 3 GPa, flexural rigidity of 2.8 GPa, and a density of 1420 kg/m^3^. A nylon filament (Skyper mono transparent filament, Amazon, Italy) of 0.33 mm thickness and with an 18 kg maximum load capacity was used for pulling the module. The rotation of the module is supplied by DC gear-motors and a gear train mechanism obtained with two spur-gear pinion and a gear (G-2), with a gear ratio of 1:2.8. The motors used for actuation are Pololu 986.41:1 micro metal gear motors (Pololu Inc., Las Vegas, NV, USA) which provide 9 kg-cm torque, 32 RPM at 12 V. 

**Figure 3:**
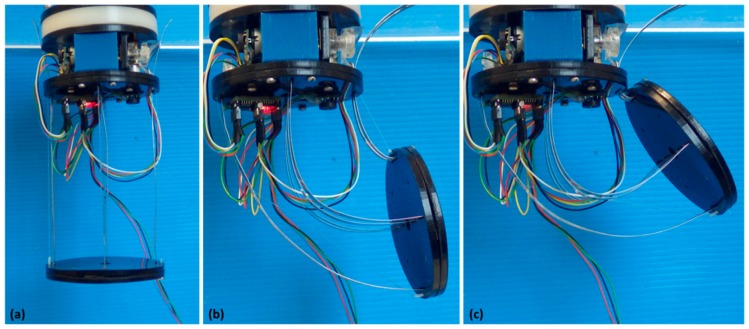
Assembled module at different bending angles. (**a**) 0° mode; (**b**) 90° mode; (**c**) 143° mode, where 143° represents the maximum bending angle achievable.

The components were fabricated by a CO_2_ laser cutting machine (VersaLaser, Universal Laser Systems, Scottsdale, AZ, USA) and assembled by classical fastening techniques. The fully assembled design of the module is shown in Figure 3 at different bending angles. 

The module hosts its own low-level controller composed of a customized board (23 × 20 mm) embedding a microcontroller (PIC32MX150F128B, Microchip Inc., Chandler, AZ, USA), two motor drivers (LV8548MC, ON Semiconductor, Phoenix, AZ, USA) to drive the three motors of the module, and an RS232 bus to communicate with a customized control user interface (developed in VB.NET, Microsoft Inc., Redmond, WA, USA) hosted on a personal computer (PC). Motors are controlled in position and speed by means of magnetic encoders (Magnetic Encoder 12 CPR, Pololu Inc.) installed on the three motors.

### 2.3. Kinematic Modeling

In order to characterize the module movements, we modeled its kinematics. Given that we wanted to maintain constant curvature, we adopted Denavit–Hartenberg (D–H) parameters (Table 1) and a transformation matrix for the kinematic analysis, following Jones et al. [25,26,27]. A constant curvature can be maintained thanks to planar spring properties of compliancy, the constant Young’s modulus, and the moment of inertia.

Here, we assumed that the *i*th link is attached to the *i*th frame in accordance with Craig’s approach [28].

**Figure 4:**
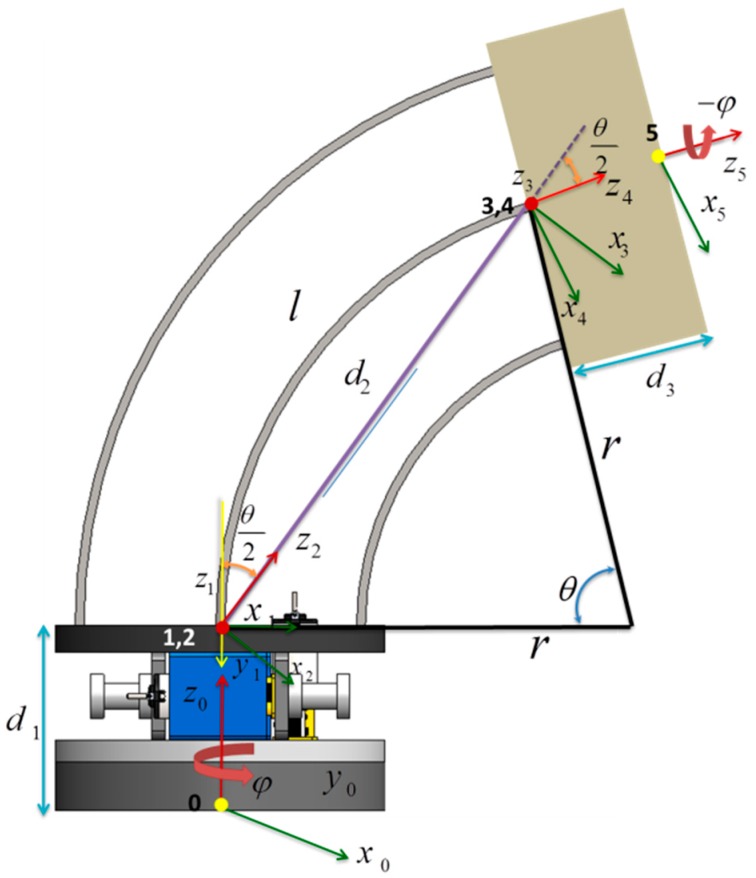
Denavit–Hartenberg parameter representation for kinematic modeling. *l* = length of the flat springs; *φ* = rotation of the module about the *z*-axis; *θ* = bending angle of the module; *r* = radius of the arc; $d_{1}$ = height of the actuator module; $d_{2}$ = translational length of the prismatic joint; $d_{3}$ = thickness of the upper plate of the bending module.

To define the D–H parameters of the arm module, we exploited the constant curvature sections of the continuum arm (see Figure 4). The evolution between one end of the module to the other can thus be described by five discrete transformations related to five virtual joints. There are three joints for bending (rotation ($\frac{\theta }{2})$, translation ($d_{2})$, and rotation ($\frac{\theta }{2})$), and two joints for axis rotation of the module (*ϕ*) for alignment with the bending plane. The results of this analysis are shown in Table 1. The final transformation matrix and end point Cartesian coordinates are reported in Equations (1) and (2), respectively.

(1) $$T_{0}^{5}=[\begin{array}{cccc} \sin^{2}\left(\varphi \right){\;+\;\cos}^{2}\left(\varphi \right)\cos\left(\theta \right) & \sin\left(\varphi \right)\cos\left(\varphi \right)\left(\cos\left(\theta \right)-1\right) & \cos\left(\varphi \right)\sin\left(\theta \right) & \cos\left(\varphi \right)\left(d_{3}\sin\left(\theta \right)+\;2\frac{l}{\theta }\sin^{2}\left(\frac{\theta }{2}\right)\right)\\ \sin\left(\varphi \right)\cos\left(\varphi \right)\left(\cos\left(\theta \right)-1\right) & \cos^{2}\left(\varphi \right){\;+\;\sin}^{2}\left(\varphi \right)\cos\left(\theta \right) & \sin\left(\varphi \right)\sin\left(\theta \right) & \sin\left(\varphi \right)\left(d_{3}\sin\left(\theta \right)+\;2\frac{l}{\theta }\sin^{2}\left(\frac{\theta }{2}\right)\right)\\ -\cos\left(\varphi \right)\sin\left(\theta \right) & -\sin\left(\varphi \right)\sin\left(\theta \right) & \cos\left(\theta \right) & d_{1}{\;+\;d}_{3}\cos\left(\theta \right)+\;2\frac{l}{\theta }\sin\left(\theta \right)\\ 0 & 0 & 0 & 1 \end{array}]$$

(2) $$\begin{array}{c} x=\cos\left(\varphi \right)\left(d_{3}\sin\left(\theta \right)+2\frac{l}{\theta }sin^{2}\left(\frac{\theta }{2}\right)\right)\\ y=\sin\left(\varphi \right)\left(d_{3}\sin\left(\theta \right)+2\frac{l}{\theta }sin^{2}\left(\frac{\theta }{2}\right)\right)\\ z=d_{1}+d_{3}\cos\left(\theta \right)+2\frac{l}{\theta }\sin\left(\theta \right) \end{array}\}$$

where (*x*, *y*, *z*) is the coordinate of the module at point 5 (see the Figure 4), l is the length of the central spring, $d_{1}$ is the height of the actuator module, $d_{2}$ is the translational length of the prismatic joint, $d_{3}$ is the thickness of the upper plate of the bending module, θ is the bending angle of the module, and φ is the rotation of module about the *z*-axis.

Mapping between actuator space and joint space is required in order to obtain the module position in function of the actuator movements. Assuming that the two tendons used for bending are driven in such a way as to avoid any compression in the module (one pulled and the other released accordingly), the module position can be correlated to the tendon lengths through the number of rotations of the pulleys ($n_{1}$, $n_{2}$). 

Keeping the constant curvature assumption, we can calculate the bending angle θ and radius of curvature *r* using the length of the central and lateral springs ($l_{s1}$,$l_{s2}$), and also a relation between the central spring length and both the lateral spring lengths.

(3) $$\{\begin{array}{c} l=r\theta \;\;\;\;\;\;\;\;\;\;\;\;\;\\ l_{s1}=\left(r+d\right)\theta \\ l_{s2}=\left(r-d\right)\theta \end{array}$$

Solving Equation (3), we obtain

(4) {l=ls1+ls22  θ=ls1−ls22d r=2ldls1−ls2

where *d* is the disk radius.

**Figure 5:**
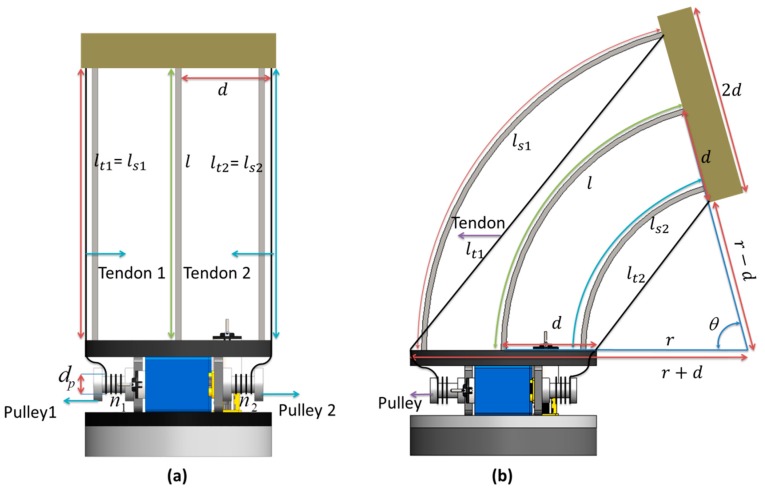
Actuator space modeling. (**a**) Module initial position at *θ* = 0°; $l=l_{t1}=l_{t2}=l_{s1}$=$\;l_{s2}$. (**b**) Bending at *θ*; *r* = radius of curvature, $d_{p}$ = diameter of the pulley, *d* = radius of the disk, $l_{s1}$,$l_{s2}=$ lengths of the lateral springs,$\;l_{t1}$,$l_{t2}$ = lengths of the tendons, $n_{1}$ = number of rotations of pulley 1,$\;n_{2}$ = number of rotations of pulley 2.

From Figure 5b, we can obtain tendon lengths $l_{t1}$ and $l_{t2}$ as in Equation (5) and length variation $\Delta l_{t1}$ and $\Delta l_{t2}$ as in Equation (6). The shorter/internal tendon (tendon 2 in Figure 5a,b) performs the bending. The longer/external (tendon 1) follows the outer lateral spring and is used to tune the stiffness.

(5) $$\{\begin{array}{c} l_{t1}=2\left(r+d\right)sin\left(\frac{\theta }{2}\right)\\ l_{t2}=2\left(r-d\right)sin\left(\frac{\theta }{2}\right) \end{array}$$

(6) {Δlt1=lt1−l=n1πdp Δlt2=lt2−l=n2πdp

Solving the system of equations, we obtained θ as

(7) $$\theta =2sin^{-1}\left(\frac{\pi d_{p}\left(n_{1}-n_{2}\right)}{4d}\right)$$

where $d_{p}$ is the diameter of the pulley.

### 2.4. Cantilever Beam Modeling 

**Figure 6:**
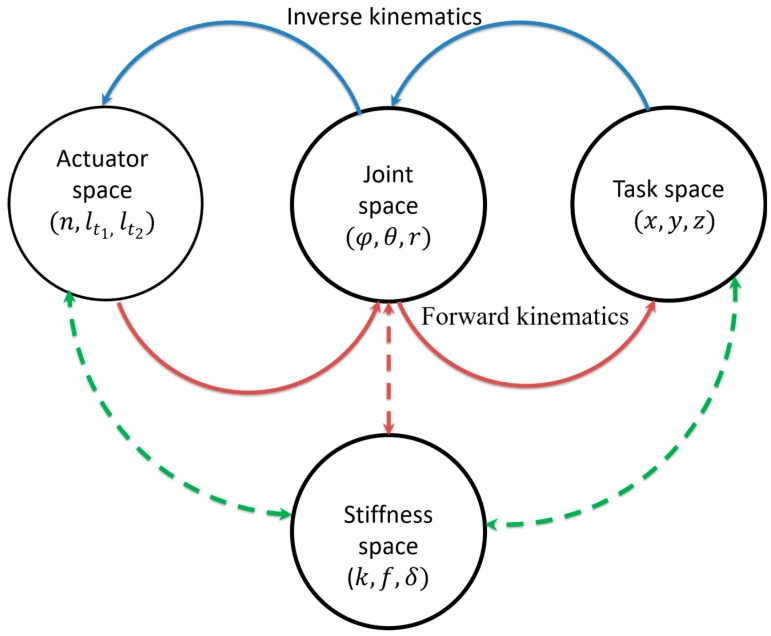
Mapping between stiffness, actuator, joint, and task space. n = number of rotations of the motor; $l_{t1}$,$l_{t2}$ = lengths of the tendons; φ = rotation of the module about the *z*-axis; *θ =* bending angle of the module; *r* = radius of curvature; (x, y,z) = end-effector coordinate points;  k = stiffness of the beam; f = bending force; δ = deflection of the module.

We modeled the arm module in the static force domain to map the stiffness of the beam (*k*) with the actuator, joint, and Cartesian space (Figure 6) by developing a relationship between the bending force and differential tendon length. This enables a stiffness-based control architecture to be developed. In fact, the deflection angle can be obtained, and the results can be compared with the kinematic model in order to validate this approach for control purposes. A model on stiffness space can also be used to help in the selection of spring dimensions (length, width, and thickness), and the best number of springs for the application desired. 

**Figure 7:**
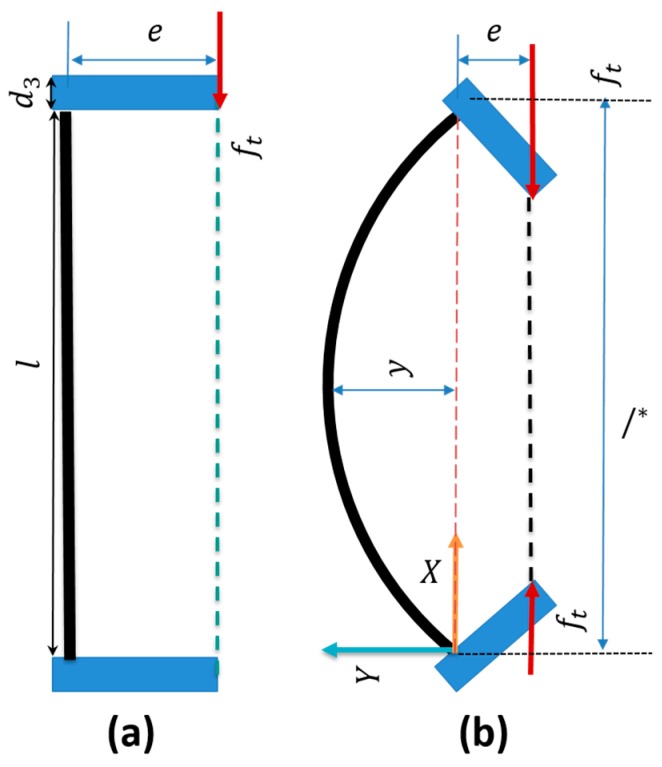
Free body diagram. (**a**) The beam in the initial position and (**b**) bent. l = length of the beam; e = eccentricity from the center; $d_{3}\;$ = thickness of the upper plate of the bending module; $f_{t}$ = bending force; y = deflection of the beam; $l^{*}$ = variable length of the beam.

Again, we assumed a constant curvature of the beam with a large deformation of the spring [29,30]. Beams are created with spring steels with a constant Young’s modulus (*E*) and an area moment of inertia (*I*) along the structure. Figure 7a,b, shows the free body diagram of the module with tendon pulling force $f_{t}$ and bending angle θ. Considering the large deformation, we used a Euler–Bernoulli simplified model, calculating the deflection and angle of the spring (see Equation (8)).

(8) $$E\cdot I\cdot \frac{d^{2}y}{dx^{2}}=M$$

where, *E* is the Young’s modulus, $I=bh^{3}/12$ is the moment of inertia with *b* the width and *h* the thickness of the planar spring, and *y* is the beam deformation. The total moment on the beam (*M*) can be calculated using Equation (10), from which we can obtain a direct relation between the deflection and pulling force ($f_{t}$).

(9) $$M=-f_{t}\left(y+e\right)$$

where *e* is the eccentricity from the center of the beam. Substituting Equation (9) into Equation (8), and considering that 1/r=θ/l (in the continuous curvature hypothesis), at the end we can get a direct relation between the curvature radius, tendon force, and deflection as a form of second order ordinary differential equation (ODE)

(10) $$E\cdot I\cdot \frac{d^{2}y}{dx^{2}}+f_{t}y=-f_{t}e$$

To solve Equation (10), we assumed the maximum deflection occurring in the middle of the beam (l2) due to the maximum bending moment and we fixed y=0  at  x=0,  y=0  at  x=l2 as boundary conditions. This way, we obtain a relation between force $f_{t}$ and deflection

(11) $$y=e\;\left(\tan\left(\sqrt{\frac{f_{t}}{EI}}\;\frac{l}{2}\right)\sin\left(\sqrt{\frac{f_{t}}{EI}}\;x\right)+\cos\left(\sqrt{\frac{f_{t}}{EI}}\;x\right)-1\right)$$

To generalize Equation (11), we considered a variable length $l^{*}$ of the beam and we obtained the maximum deflection as

(12) $$y_{max}=e\;\left(\sec\left(\sqrt{\frac{f_{t}}{EI}}\;\frac{l^{*}}{2}\right)-1\right)$$

From Equation (12) we can obtain the tendon pulling force as

(13) $$f_{t}=E\cdot I\cdot {\left[\frac{2}{l^{*}}\cos^{-1}\frac{e}{y_{max}+e}\right]}^{2}$$

where $l^{*}$ is the distance between the two beam ends for maximum deflection $y_{max}$. After getting $f_{t}$, we obtain the module’s bending stiffness as

(14) $$k=\frac{f_{t}}{\Delta l}$$

**Figure 8:**
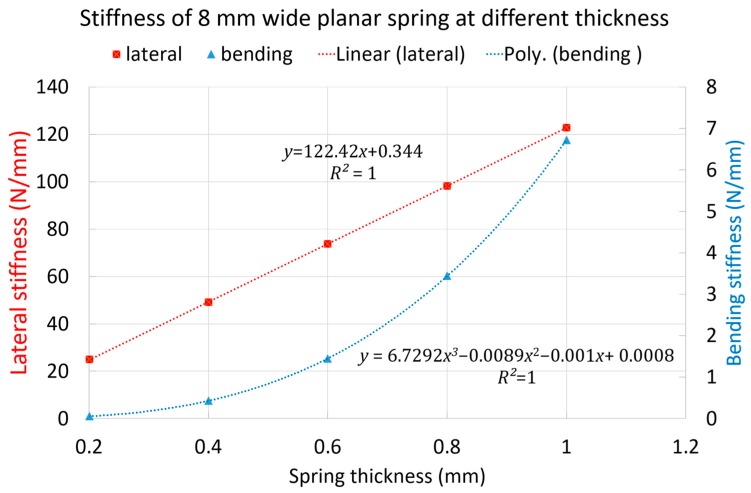
Lateral and bending stiffness of the module for different thicknesses. Poly: third degree polynomials.

From Equations (12) and (13), we mapped the deflection and force and using Equation (14) we calculated the bending stiffness at different thicknesses of the 8 mm wide planar spring. We found that in the bending direction, the stiffness varies with a third-degree polynomial. On the other hand, the lateral stiffness, based on the point load cantilever beam model with infinitesimal deformation, varies linearly at different thicknesses (Figure 8). The thicker the spring, the stiffer and stronger the module is, the thinner the spring is, and the more compliancy is achieved. 

### 2.5. Experimental Setups 

**Figure 9:**
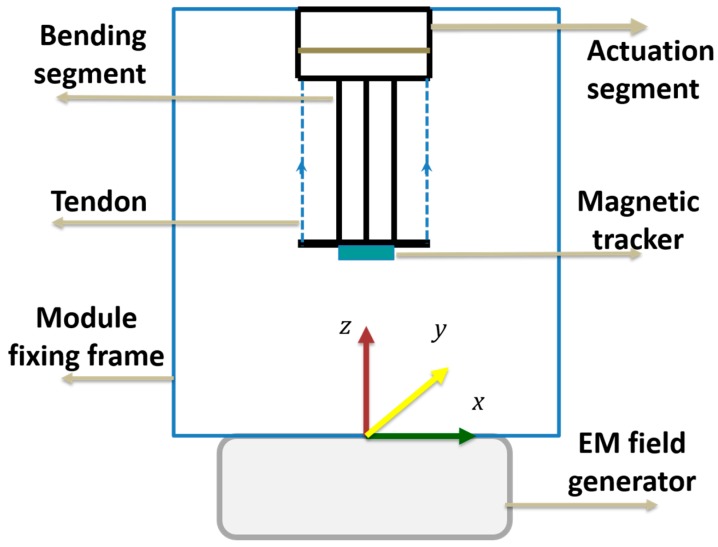
Aurora system. Setup to measure the position and orientation of the module. EM: Electromagnetic.

We used NDI’s Aurora Technology (Northern Digital Inc., Ontario, Canada) for the workspace analysis. This consists of an electromagnetic (EM) field generator, sensor and sensor data amplifier. The Aurora system can record the data regarding positions (T*x*, T*y*, T*z*) and orientation (R*x*, R*y*, R*z*) at a 40 Hz rate with an accuracy of 0.8 mm and 0.70°. We fixed the module in the machine frame, placing the sensor on the tip of the module. Starting from a straight position, we applied a constant pulling speed to the tendon up to the maximum bending angle, recording the angular positions, Cartesian positions, and number of rotations of the motor in real time using a motor encoder (Figure 9). The experiments were repeated 10 times.

**Figure 10:**
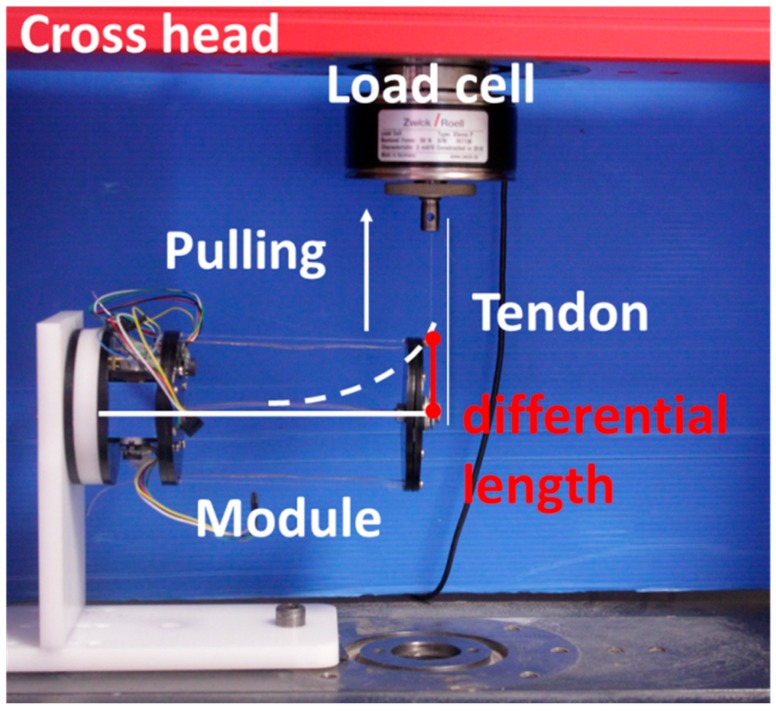
Experimental setup for stiffness measurement. A tendon connects the end of the module with the load cell. By pulling this tendon, the pulling force is measured, and the stiffness is obtained with a fixed length of module tendons without their actuation. The distance traveled is the differential length, or deflection, of the module.

To characterize the bending and lateral stiffness of the module, we used a Universal Testing Machine (UTM, Zwick/Roell Z005, Zwick GmbH, Ulm, Germany) to pull the module and to acquire the forces (See Figure 10). The forces generated were measured by a load cell of ±1 kN with 0.001% of resolution.

## 3. Results

**Figure 11:**
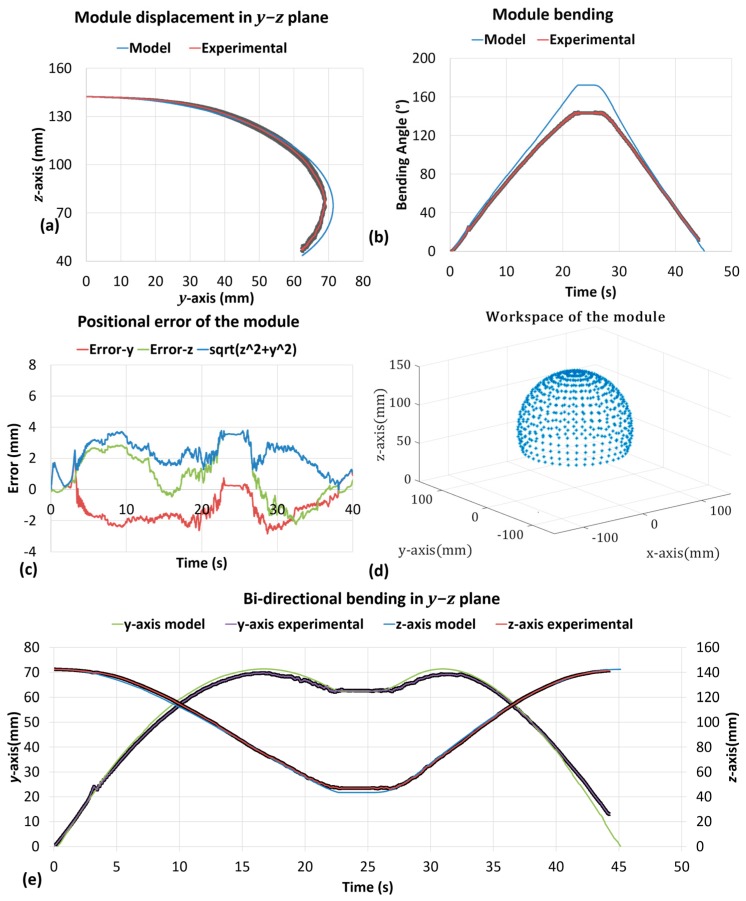
Kinematic model validation and characterization of the module. (**a**) Bending of the module in *x*–*y* plane; (**b**) model validation of the bending angle about *x*-axis; (**c**) measured error in *y*–*z* plane between Aurora results and model; (**d**) whole workspace of the module in 3D space; and (**e**) bi-directional bending of the module.

Positional measurements acquired with the Aurora system were compared with the output of the kinematic model (Figure 11a). The maximum length along the *z*-axis (142.56 mm) corresponds to the straight position. The minimum length (46.32 mm) corresponds to the maximum bending angle achieved by the module (143.23°). Figure 11b,c shows the bending and returning behavior of the module, demonstrating the effectiveness of the model predictions obtaining at maximum a positional root mean square error (RMSE) of 3.80 mm that corresponds to 6.3% of the module diameter. Figure 11d shows the 3D working space with constant radius about its rotation axis of the module which can be achieved with the *z*-axis rotation at different bending angles. Figure 11e shows the bi-directional bending in the *y*–*z* plane which mapped with experimental results and model.

**Figure 12:**
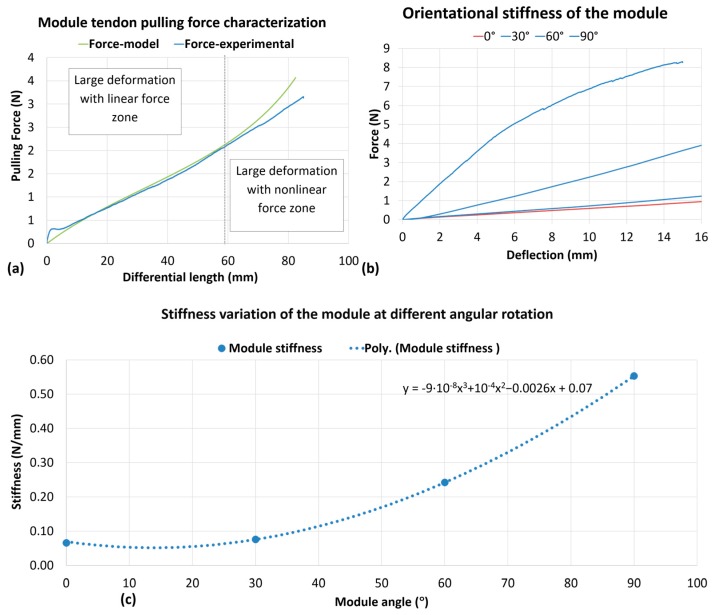
Force and stiffness characterization of the module at different angles. (**a**) Tendon pulling force and model prediction; (**b**) measurement of bending force; (**c**) module stiffness behavior as a function of module positional angle with polynomial fitting. Poly: Third order polynomials.

The results of the pulling force experiments were compared with our static model which was shown to fit the experimental data up to 65 mm of differential length reaching ≈100° bending (Figure 12a). The small deviation obtained after 65 mm can be reduced with a closed loop control and by adopting more precise manufactured components in the arm module. 

We also evaluated the stiffness of the module, measuring forces in 15 mm of deflection at different positions along the circumference of the module’s external disk. The module was rotated with respect to its central axis for 0° corresponding to the bending plane, 30°, 60°, and 90° corresponding to the lateral plane; obtaining 0.066 N/mm for 0°, and 0.088, 0.25 and 0.55 N/mm for 30°, 60°, and 90°, respectively (see Figure 12b,c). From the results, we estimated that the stiffness would increase with cubic polynomials with the highest value, 90°, at 8.33 times higher than the lowest value, which is at 0°.

## 4. Discussion

We have presented the design and characterization of the basic module for a modular continuum arm which can perform manipulation in unstructured environments. The key feature of the single module is its internal modularity, with separate operative and control units (see Figure 2). All the service components (motors, encoder, and controller) are transferred to the control unit, thus improving the controllability with respect to most state-of-the-art arms, where actuation is located at the base in order to take advantage of the joint of inertia at the expense of control complexity [18,25,31,32].

The operative unit, actuated with a tendon-motor mechanism, contains planar springs that can bend only in one plane, with the advantage of high passive and constant stiffness on the perpendicular plane. In our case, we measured over eight times higher perpendicular stiffness than the stiffness recorded in the bending plane. This feature is fundamental in guaranteeing constant inertia and a high robustness of the system. In addition, using planar springs, the system can support movement without buckling or lift weight without any heavy stiffening mechanism. Our arm can transversely lift 8 N without any actuation and can be improved by simply changing the module’s central spring thickness or width or both by increasing the number of springs.

We have shown that by combining bending and rotational joints, a large deformation (≈143°) and a full working space (360°) can be achieved by a single module. This high deformability, which cannot be achieved with rigid arms, enhances the module adaptation and could lead to developing a modular arm capable of accomplishing difficult tasks requiring high compliancy with the environment, e.g., passing through a series of obstacles and gates. Our system shows two strategies for stiffening each module. The first strategy increases the stiffness by using a combination of planar springs with module rotation, while the second one exploits the antagonistic control of the tendons’ actuation. The combination of these two mechanisms allows the complete stiffening of the module. During the characterization experiment, the module also showed the advantage of repeatability due to spring behavior (simply pulling in elastic limit) and joint rotation in order to acquire the full workspace. Examples of bending and rotation of the module are presented in the Supplementary Video S1. This could lead to a reliable solution for precise unstructured manipulation tasks as demonstrated in the paper through the experimental and theoretical approach.

**Figure 13:**
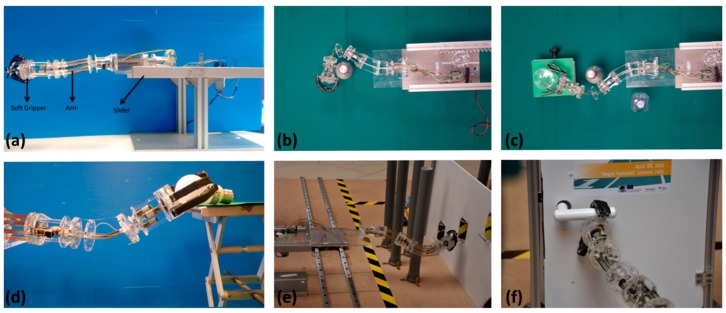
First prototype of SIMBA and its capabilities at different tasks. (**a**) Full assembled design of the manipulator [16]; (**b**) bending capability; (**c**) dexterity; (**d**) capacity to bend and rotate for manipulation task; (**e**) arm positioning; (**f**) door opening.

In a previous work [16], we demonstrated the potential to make a modular arm that exploits the basic module described (See Figure 13a). Our laboratory tests revealed the high flexibility of the SIMBA manipulator to configure itself for object-oriented grasping or pouring water (see Figure 13b–d). In addition, during the first International Soft Robotic Challenge 2016 [33], SIMBA performed real-time tasks such as door opening and obstacle avoidance (see Figure 13e,f). 

## 5. Conclusions

In the current work, we have presented kinematic and cantilever beam models to describe the positions and stiffness of the basic module for a modular continuum arm, respectively. The kinematic model demonstrates its accuracy and repeatability (Figure 11) with only an error at a maximum of ≈6% in the module diameter. In both models we used the constant curvature assumption and adopted the principle of the Euler–Bernoulli beam theory for large deformation. We thus demonstrated that the cantilever beam model has a good prediction regarding experimental data of up to ≈100° of bending. The nonlinearity obtained by the experimental results for bending greater than 100° can be attained by reciprocal mapping of kinematic and stiffness domains, thus also facilitating the implementation of more precise control strategies.

The proposed module is very versatile and can easily act not only as arm component but can be integrated with all applications where directional stiffness, compliancy, rotational, and bending capabilities are required. For instance, alternative applications of the module could be as a leg for an alligator-inspired robot where stiffness is required in the plane perpendicular to the motion, and the inherent compliance of the module could be exploited for unstructured terrains [34,35]. Similarly, the module could be adopted for snake-inspired robots, where directional stiffness and axis rotation can be exploited for different snake locomotion modalities (sidewinding, corkscrewing, and strafing) [36,37,38,39]. 

The high compliancy and robustness demonstrated by the module, and even by an entire arm based on the presented design, also opens several other opportunities for applications in unstructured environments, for pipe inspections, or manipulation in hazardous environments. 

## Figures and Tables

**Table 1:** Denavit–Hartenberg (D–H) parameters of the single SIMBA arm module.

Link (*i*)	*α*	*a*	*d*	ϑ
1	$-\frac{\pi }{2}$	0	$d_{1}$	φ
2	$\frac{\pi }{2}$	0	0	$\frac{\theta }{2}$
3	$-\frac{\pi }{2}$	0	$d_{2}$	0
4	$\frac{\pi }{2}$	0	0	$\frac{\theta }{2}$
5	0	0	$d_{3}$	−φ

*α =* link twist; *a* = link length; d = link offset, $d_{1}$ = height of the actuator module; $d_{2}$ = translational length of the prismatic joint; $d_{3}$ = thickness of the upper plate of the bending module; ϑ = joint angle; *θ* = module bending angle; φ = rotation of the module about the *z*-axis.

## Supplementary Materials

The following is available online at http://www.mdpi.com/2313-7673/3/1/3/s1, Video S1: Modular continuum manipulator: Analysis and characterization of its basic module.
